# Optimizing Large-Volume Paracentesis in a Gastroenterology Ward: A Two-Cycle Quality Improvement Project

**DOI:** 10.7759/cureus.104453

**Published:** 2026-02-28

**Authors:** Lok Man Chuen, Aditi Sinha, Salonee Dhungana, Inez McCurley, Rehan Khan

**Affiliations:** 1 Gastroenterology, London North West University Healthcare NHS Trust, London, GBR

**Keywords:** ascitic drain, documentation improvement, gastroenterology, large-volume paracentesis, procedural efficiency, quality improvement

## Abstract

Background

In a busy gastroenterology ward in a London district general hospital, delays were frequently encountered when preparing large-volume paracentesis, mainly due to difficulties in locating and gathering equipment. This Quality Improvement Project (QIP) aimed to standardize equipment preparation, improve perceived efficiency, and enhance post-procedure documentation.

Methodology

A resident doctor-led, two-cycle QIP was conducted using the Plan-Do-Study-Act (PDSA) methodology. Cycle one focused on equipment preparation. Baseline data were collected via surveys, followed by the introduction of a standardized equipment checklist poster displayed at the doctors’ office. Self-reported time taken to locate and gather equipment was collected pre- and post-intervention. Cycle two focused on post-procedure documentation through the development of a smart text proforma within the electronic patient record to promote safe and consistent documentation. Self-reported time taken for documentation and user feedback was collected.

Results

There were nine respondents before and six after the poster intervention in cycle one, while in cycle two, there were eight respondents before and six after the introduction of the smart text proforma. At baseline, over half of the resident doctors (five out of nine) required 6-10 minutes to prepare equipment, and 78% (seven out of nine) reported incomplete knowledge of required items. Following the poster intervention, all six respondents had seen the poster, and 83% (five out of six) felt it reduced preparation time. In cycle two, baseline data showed that 50% (four out of eight) required guidance for documentation, with 38% (three out of eight) spending four to six minutes and 25% (two out of eight) spending over 10 minutes per note. After introducing the proforma, all six respondents found it helpful and anticipated being able to complete documentation independently. Overall, 50% (three out of six) and 33% (two out of six) anticipated spending one to three minutes and four to six minutes per note, respectively. Qualitative feedback highlighted clearer and faster documentation.

Conclusions

Overall, the standardized equipment checklist and smart text proforma were perceived to reduce procedural delays and were reported to improve documentation consistency. They also improved trainee confidence and reduced uncertainty. Similar approaches could be applied to other departments and procedures, with local refinement to ensure ongoing accuracy and relevance.

## Introduction

Large-volume paracentesis (LVP) is a common but time-sensitive ward-based procedure for the management of symptomatic ascites in patients with liver disease and other conditions [1]. It involves multiple steps and requires familiarity with specific equipment. Clear and comprehensive documentation of consent, procedural details, and post-procedure care is essential to ensure patient safety and support continuity of care. International and specialty guidelines, such as those from the British Society of Gastroenterology (BSG), emphasize the importance of consistent practice and documentation for invasive procedures, including the BSG eight-point checklist for LVP [2].

In a busy gastroenterology ward in a London district general hospital, preparation of equipment and documentation is performed by several resident doctors who rotate regularly, so new doctors may be unfamiliar with ward-specific equipment and processes. Verbal feedback from resident doctors highlighted repeated delays when preparing for ascitic drain insertion, predominantly due to difficulty identifying, locating, and gathering the required equipment, especially at the start of new rotations. The lack of a consistent, easily accessible, and updated equipment list was identified as a potential contributor.

In addition, clinicians have individual preferences in post-procedure documentation, and some doctors require senior input to ensure notes are complete and safe. These factors are perceived to contribute to further delays and inconsistencies in clinical records. Audits of abdominal paracentesis have identified gaps in quality measures, including inconsistent documentation of key elements such as consent and post-procedure care [3]. Evidence from quality improvement projects (QIPs) in similar procedural settings suggests that simple standardization measures, such as formalized equipment lists, checklists, and structured documentation, can improve safety, efficiency, staff confidence, and adherence to guidelines [3-5].

This resident doctor-led QIP aimed to improve both the efficiency and consistency of ascitic drain preparation and documentation and is reported in accordance with the Standards for QUality Improvement Reporting Excellence (SQUIRE) 2.0 guidelines. Specifically, the project sought to improve workflow efficiency and reduce perceived delays for resident doctors in gathering the required equipment, and to improve the quality and efficiency of post-procedure documentation through the introduction of standardized interventions. These help reduce avoidable delays, support trainee growth, and improve the quality of patient care.

## Materials and methods

This QIP was conducted in the Gastroenterology Department at Ealing Hospital, part of London North West University Healthcare NHS Trust, London, United Kingdom. Cycle one was conducted from September 17, 2025, to October 26, 2025, and cycle two from November 10, 2025, to December 2, 2025. Data were collected anonymously using structured questionnaires distributed to the nine eligible gastroenterology resident doctors at baseline and after each intervention; locum doctors were also involved where applicable. Baseline data were obtained before the introduction of the equipment checklist poster (Appendix A) and documentation smart text (Appendix B), with follow-up data collected after each Plan-Do-Study-Act (PDSA) cycle to assess change.

The survey questionnaires were developed by the resident doctor project team in line with the aims of each PDSA cycle. The pre-intervention questionnaire for cycle one (Appendix C) identified barriers to efficient equipment preparation and assessed interest in a standardized equipment checklist poster, including perceived usability. The post-intervention questionnaire (Appendix D) evaluated poster visibility, accessibility, and perceived usefulness. For cycle two, the pre-intervention questionnaire (Appendix E) established baseline self-reported documentation time and explored barriers to clear post-procedure documentation. The post-intervention questionnaire (Appendix F) assessed the usefulness of the smart text proforma, its perceived impact on efficiency, self-reported documentation time when using the proforma, and whether it reduced the need for senior guidance. Formal psychometric validation was not undertaken as this was a QIP. Face validity was ensured through review and approval by the supervising consultant before implementation. However, the questionnaires were reviewed to ensure they were appropriate and covered all key areas relevant to the project objectives.

Senior gastroenterology clinicians were consulted throughout to ensure clinical accuracy and acceptability of the interventions, and registrar feedback on equipment preferences informed checklist refinement. Patients and the public were not involved, no patient-identifiable data were used, and the project was exempt from institutional review board approval.

PDSA cycle one: equipment preparation for ascitic drains

The primary outcome measure for PDSA cycle one was the self-reported time taken by resident doctors to identify, locate, and gather the equipment required for an ascitic drain. This was defined as the self-reported time, in minutes, from starting to gather equipment to being ready to perform the procedure. Doctors’ self-reported knowledge of the required equipment and its location was included as a secondary outcome measure. These measures were chosen as they directly reflected perceived sources of delay and were practical to collect in a busy ward environment.

Delays were thought to arise mainly from uncertainty about which items were required and where they were stored. To address this, a formalized, easily accessible, and updated equipment checklist was designed. It listed all items required for ascitic drain insertion, along with guidance on appropriate ascitic fluid investigations, and was displayed in the gastroenterology doctors’ office. The aim was to support more efficient preparation.

A follow-up survey was distributed after implementation to assess change. Data collected included self-reported preparation time, awareness of required equipment and its location, and perceived usefulness of the poster. Feedback from registrars regarding individual preferences for specific equipment prompted further revision of the checklist. The poster was actively signposted to new doctors joining the ward to encourage consistent use.

PDSA cycle two: post-procedure documentation

For PDSA cycle two, the primary outcome measure was the self-reported time taken to complete post-procedure documentation following ascitic drain insertion. Secondary measures included whether doctors needed to seek guidance from colleagues and the perceived usefulness of the smart text proforma, as well as to assess efficiency, confidence, usability, and consistency of post-procedure documentation.

Inconsistencies and delays were thought to result from uncertainty about expected documentation content rather than reluctance to document. A smart text documentation proforma was then developed within the electronic patient record system (CERNER), aligned with the BSG eight-point checklist for LVP. The proforma included consent, key procedural details, post-procedure care, and nursing instructions, aimed to reduce documentation time and improve confidence among resident doctors.

A follow-up survey was distributed to assess documentation time using the proforma and its perceived usefulness. No significant barriers to implementation were identified. To support sustainability, the smart text proforma was made accessible to all resident doctors on the gastroenterology team to encourage routine use.

## Results

Equipment preparation (PDSA cycle one)

**Figure 1:**
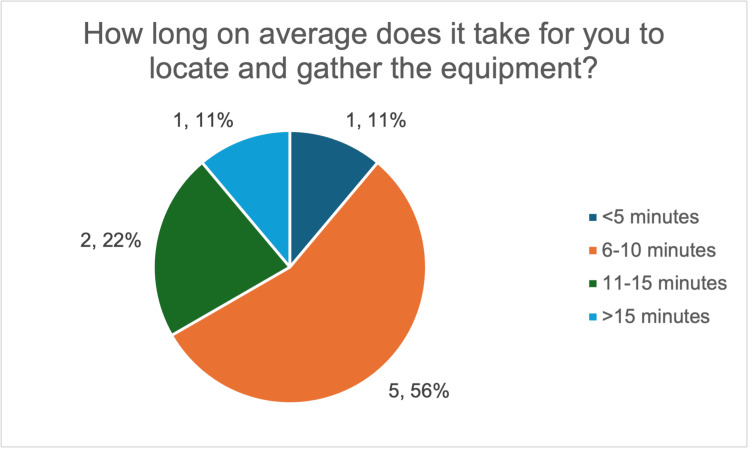
Survey responses on the average time taken by resident doctors to locate and gather equipment for large-volume paracentesis. The image was created by the authors.

**Figure 2:**
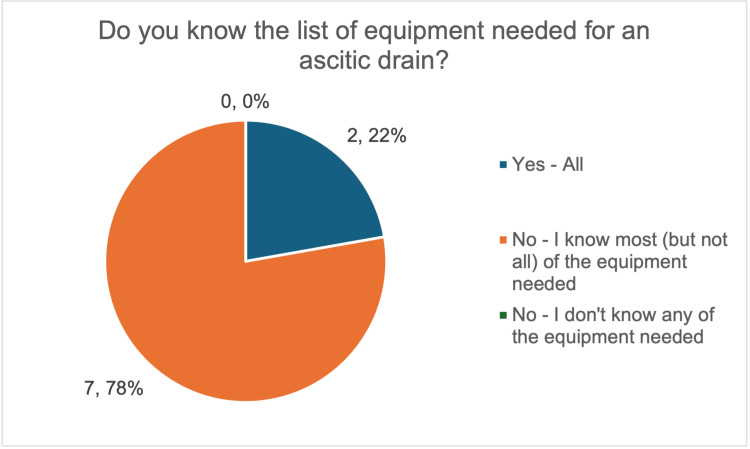
Survey responses on resident doctors’ knowledge of the equipment required for large-volume paracentesis. The image was created by the authors.

At baseline, there was marked variation in the time taken to prepare equipment for ascitic drain insertion. Most respondents (five out of nine, 56%) reported requiring 6-10 minutes, 22% (two out of nine) reported taking 11-15 minutes, while 11% (one out of nine) reported taking more than 15 minutes, demonstrating variability in preparation times; only one respondent was able to prepare equipment within five minutes (Figure 1). Moreover, 78% (seven out of nine) reported knowing most, but not all, of the required equipment (Figure 2), highlighting an incomplete knowledge of required equipment.

**Figure 3:**
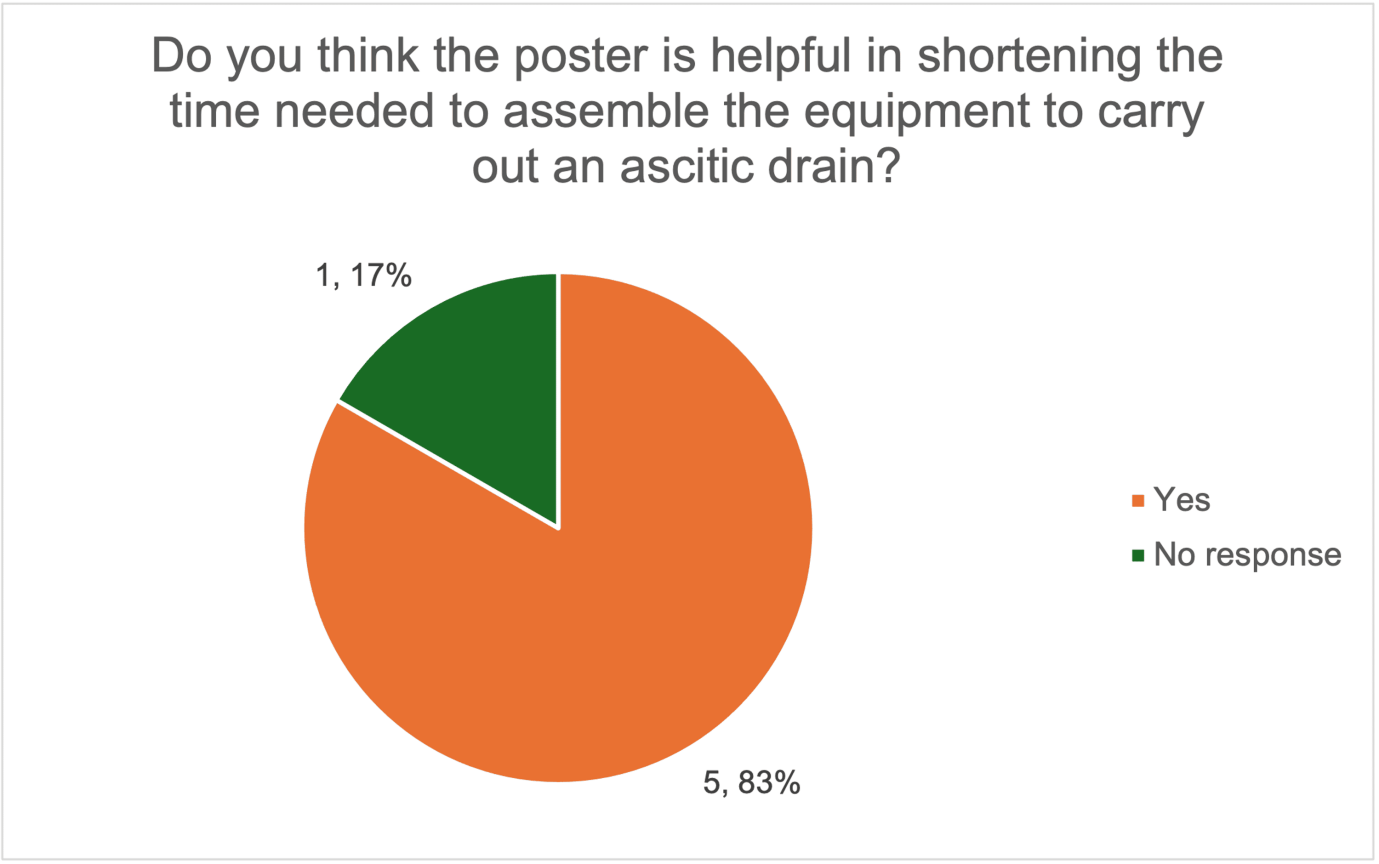
Survey responses on the perceived usefulness of the poster in reducing equipment preparation time for ascitic drains. The image was created by the authors.

After the introduction of the equipment checklist poster, all six respondents reported having seen the poster and found it useful. Most doctors (five out of six, 83%) felt that the poster helped reduce preparation time (Figure 3). Preparation time was not objectively measured; however, most respondents reported that preparation felt quicker following the introduction of the poster, reflecting perceived rather than objectively measured improvement.

Initially, differences in individual clinician preferences for equipment introduced variability. This was addressed by refining the checklist after discussion with senior clinicians to ensure accuracy. There were no delays, safety concerns, or negative impacts on patient care reported because of the intervention.

Post-procedure documentation (PDSA cycle two)

**Figure 4:**
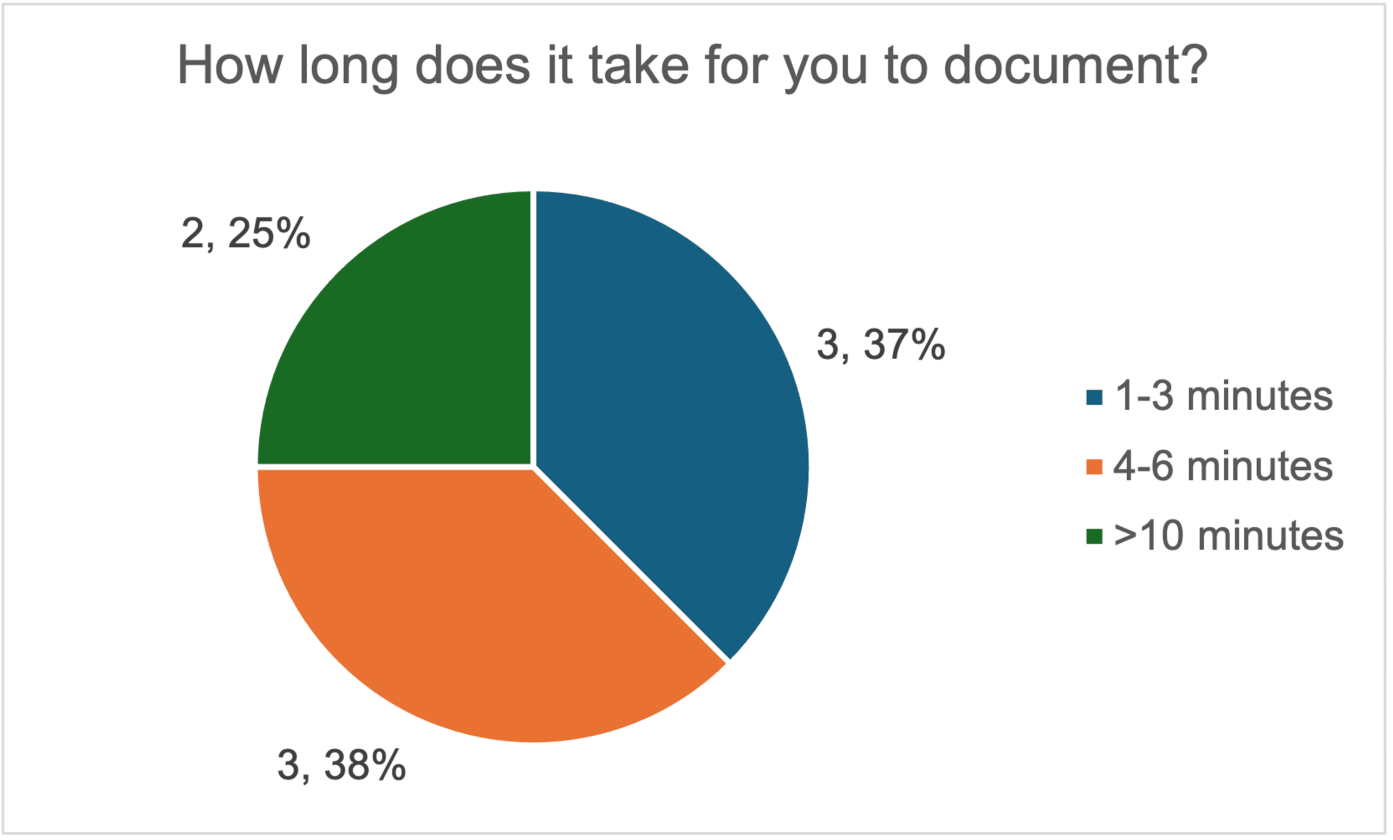
Survey responses on the time taken by resident doctors to complete post-procedure documentation for large-volume paracentesis. The image was created by the authors.

**Figure 5:**
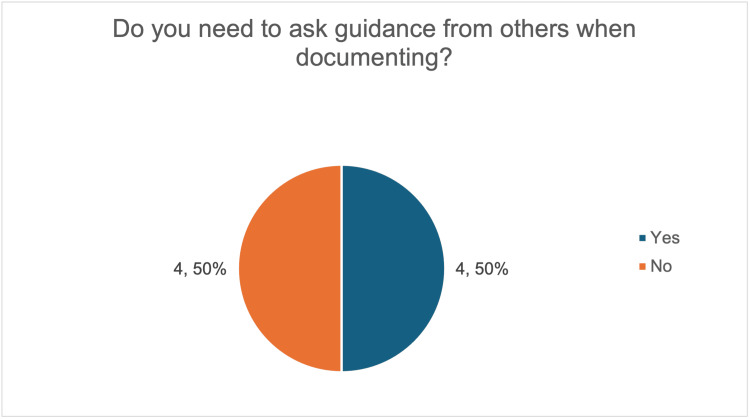
Survey responses on whether resident doctors required guidance from colleagues or seniors when completing post-procedure documentation for large-volume paracentesis. The image was created by the authors.

Baseline data demonstrated wide variation in both documentation time and confidence. Overall, 38% (three out of eight) reported spending four to six minutes completing notes, while 25% (two out of eight) reported spending more than 10 minutes (Figure 4). Half of the respondents (four out of eight) also needed guidance from colleagues to complete documentation (Figure 5).

**Figure 6:**
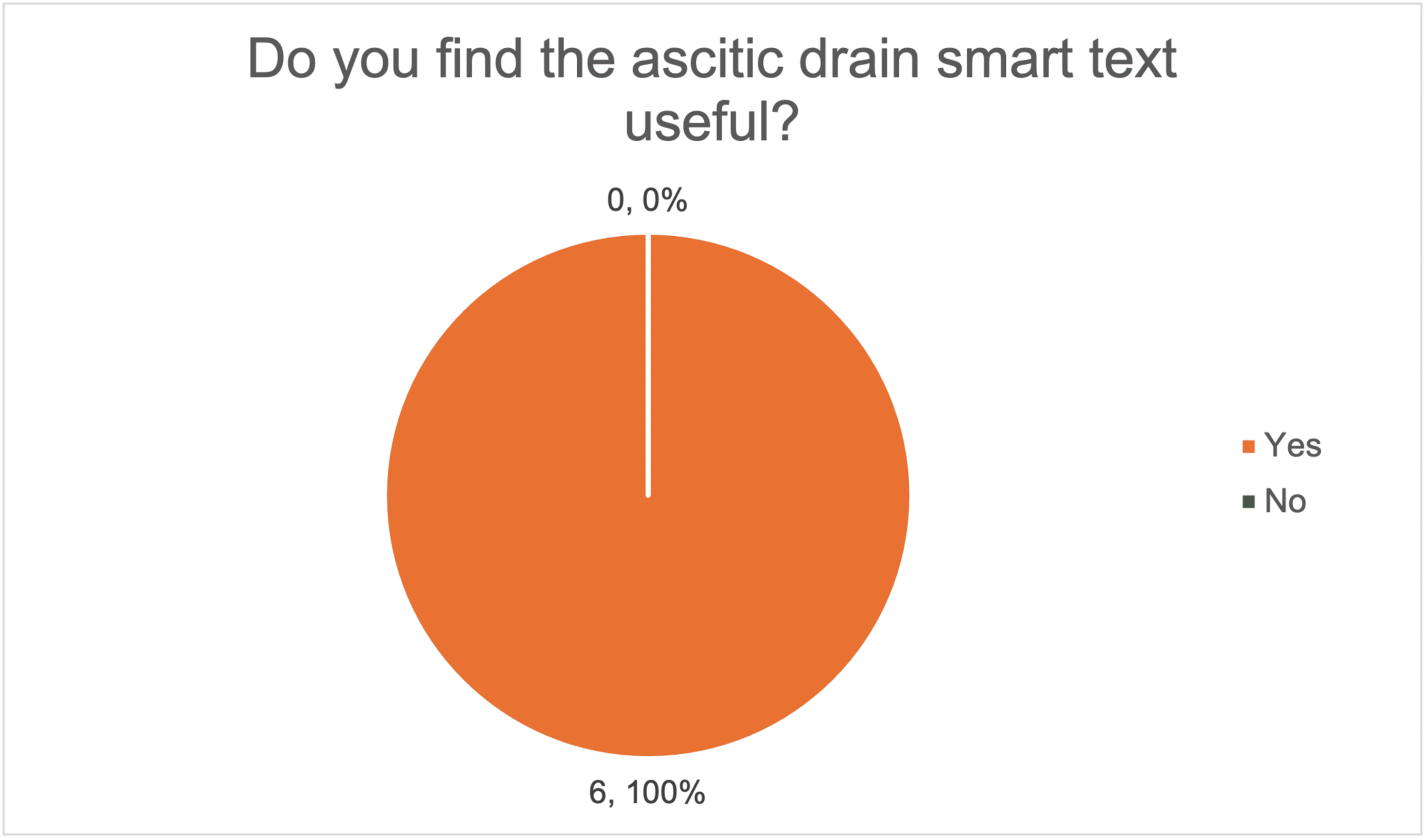
Survey responses on the perceived usefulness of the ascitic drain smart text documentation proforma. The image was created by the authors.

**Figure 7:**
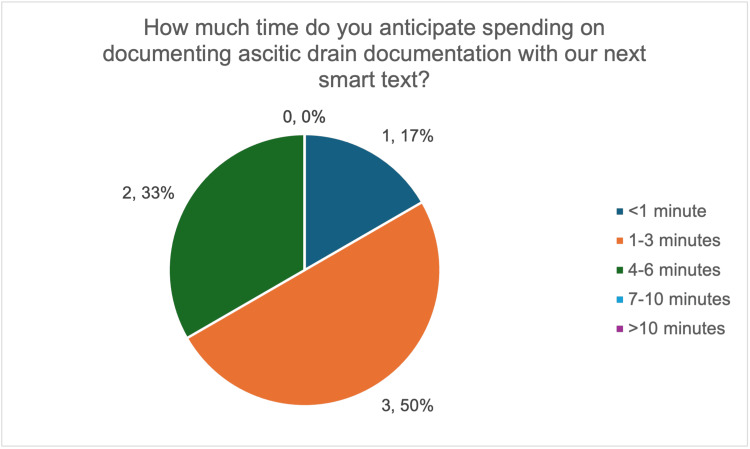
Survey responses on anticipated time to complete ascitic drain documentation using the smart text proforma. The image was created by the authors.

Following the introduction of the smart text proforma, all six respondents reported that it was useful (Figure 6) and expected it to reduce documentation time. Most respondents (five out of six, 83%) anticipated being able to complete documentation within one to six minutes, with 50% (three out of six) expecting it to take less than four minutes (Figure 7), suggesting a perceived improvement.

Respondents also reported increased confidence, with all six respondents indicating that they could independently perform post-procedure documentation. This suggested that delays were mainly due to uncertainty about what needed to be included, rather than reluctance to document.

Data completeness and unintended consequences 

Response rates were high across both PDSA cycles, although overall numbers were small due to the limited number of resident doctors in the department. Minor variation in respondent numbers occurred due to staff availability at the time of data collection.

No unintended negative consequences were identified. Anticipated benefits included improved trainee confidence and reduced reliance on senior colleagues. Both interventions were cost-effective and improved efficiency. Further areas of improvement, such as the addition of visual aids to the poster, were also identified, which will help guide future refinements.

## Discussion

This QIP highlighted unfamiliarity with local equipment and documentation requirements as one of the key contributors to delays in procedural preparation and post-procedure documentation for LVP. Our findings are consistent with the literature describing the potential benefits of standardization, such as checklists and procedural kits, in improving procedural quality and adherence to best practice. For example, Fyson et al. demonstrated that introducing a standardized paracentesis procedure checklist and equipment kit significantly improved documentation of informed consent and aseptic technique, with reduced complication rates, suggesting procedural tools can enhance both quality and safety in paracentesis practice [3].

Structured checklist interventions have a well-established evidence base across clinical domains, including procedural interventions similar to paracentesis. For example, the World Health Organization Surgical Safety Checklist has been shown to reduce morbidity and mortality and increase compliance with procedural standards [6]. Beyond surgery, checklists and bundles have improved process measures and reduced adverse events in critical care and procedural specialties, such as central line-associated bloodstream infections and bronchoscopy [7-9].

The active involvement of resident doctors in developing and refining the equipment checklist and documentation proforma in this project likely contributed to its effectiveness. Engagement of frontline clinicians in intervention design is recognized as a key driver of sustainable improvement, and it has been associated with greater engagement, improved patient safety outcomes, and more successful implementation compared with top-down approaches [10-13]. This approach aligns with broader quality improvement methodologies that emphasize iterative testing, stakeholder engagement, and co-design to accelerate the adoption and sustainability of effective tools.

By combining continuous refinement through PDSA cycles with clear alignment to SMART goals and measurable outcomes, the project employed evidence-based quality improvement methods shown to increase the likelihood of success, and the findings suggest perceived improvements following the two interventions [14]. As there is significant variability among resident doctors in performing LVP, the poster and the proforma reinforce education on what and why to send samples for analysis after the procedure, the diagnostic criteria for spontaneous bacterial peritonitis, the importance of documenting consent, early recognition of common post-procedural complications, and post-procedural checks. Structured preparation has been shown to reduce procedural stress and improve cognitive support, contributing to safer and more efficient practice [15].

Our finding of variability in baseline practice and documentation echoes observations in other QIP reports: structured templates and documentation proformas have been shown to significantly increase the completeness and consistency of clinical records. For example, standardized forms in pediatric inflammatory bowel disease increased structured documentation from 21% to 72% post‑intervention [16], while structured admission templates improved general admission note completeness [17]. Electronic templates and checklists in electronic health records have also been shown to reduce variability, improve workflow, and enhance documentation quality [15,18].

Despite these supportive findings, there were several limitations identified in this project, including a small sample size and outcomes relying on self-reported timing rather than objective measurement. No observational, audit, or electronic health record-derived data were collected. As the project was conducted on a single gastroenterology ward, the applicability to other settings may be difficult to predict. Differences between individual practices, the rotational nature of resident doctors, and changes to locations of equipment storage over time also meant that ongoing review and refinement of the interventions were necessary.

Future QIP cycles could address these limitations by expanding sample size and incorporating objective timing measures. Evaluation could also compare documentation completed with and without the smart text template, assessing inclusion of consent, procedural details, and post-procedure care, providing a more objective measure of template effectiveness. Embedding structured preparation checklists and documentation proformas into routine clinical workflows across departments may increase uptake and sustainability, as demonstrated in other internal medicine quality improvement initiatives [19]. Broader implementation and multisite collaboration could further assess generalizability and scalability, while regular review ensures tools remain accurate, up-to-date, and aligned with evolving clinical standards.

## Conclusions

Delays in procedural preparation and documentation for ascitic drains are common on busy gastroenterology wards and are often related to unfamiliarity with local equipment and documentation requirements. This QIP provides practical, ward-based evidence that simple, low-cost interventions, such as an equipment checklist poster and a smart text documentation template, can improve feasibility, usability, and perceived workflow benefit. Post-intervention data showed reduced perceived equipment preparation time and improved efficiency of post-procedure documentation. Although outcomes were primarily based on self-reported timing and perceived usefulness, consistent qualitative feedback demonstrated that both interventions were helpful, well accepted, and refined effectively through iterative PDSA cycles and user feedback. The project was cost-effective, with expenses limited to poster printing and minor IT configuration. By reducing time spent on equipment gathering and documentation, the interventions may have the potential to improve workflow efficiency and support more efficient use of clinical resources; however, formal cost or operational analysis was not performed. Sustainability has been addressed through visible poster placement, verbal signposting for new doctors, and planned updates to maintain accuracy. Overall, this project demonstrates that resident doctor-led quality improvement, focused on small and practical changes, can sustainably improve everyday clinical workflow, with potential for adaptation across other wards or institutions.

## Figures and Tables

**Figure 8:**
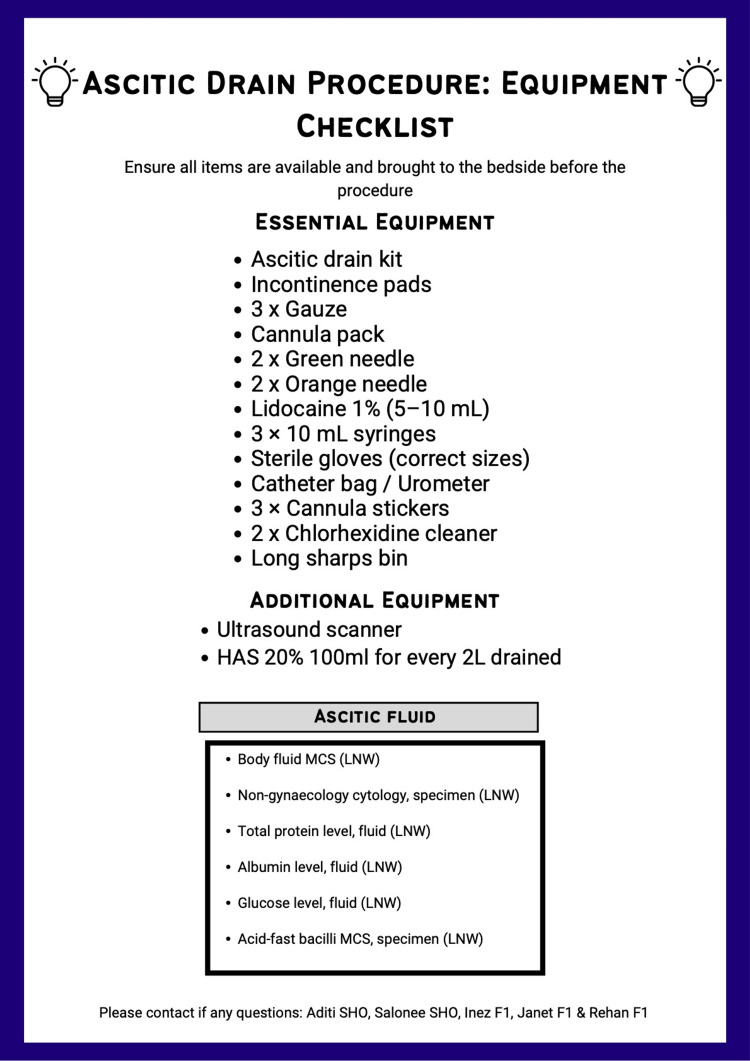
Equipment checklist for the ascitic drain procedure. The checklist was created by the authors.

## Appendices

Appendix A

Appendix B

Ascitic Drain Procedure Record

1. Indication for procedure (*please delete as appropriate*)

Diagnostic tap (for analysis) // Therapeutic drainage (symptom relief) // Both

2. Informed consent (*please delete as appropriate*)

Verbal consent // Written consent obtained

Information provided to the patient

- Explanation of the procedure, purpose, and likely benefits

Risks and Possible Complications Explained (general risks listed, but not exhaustive):

Pain or discomfort at the insertion site // Bleeding or haematoma // Infection (local or peritonitis) // Leakage of ascitic fluid from the site // Injury to abdominal organs (bowel, bladder, blood vessels) // Hypotension or dizziness // Electrolyte disturbance or renal impairment // Reaccumulation of ascites // Need for repeat procedure

3. Pre-procedure checks (*please delete as appropriate*)

- Coagulation profile checked (INR, platelets)

- Ultrasound used for site marking

- Albumin prepared (if >5L expected to drain)

- Baseline observations recorded (BP, HR, Temp, SpO_2_)

4. Procedure details

Technique: Ultrasound // Blind

Area cleaned with chloroprep

(x) mL 1% lidocaine administered locally to the insertion site

Incision made to assist insertion

Uncomplicated insertion

(description of fluids: color, etc)

6G Rocket drain inserted at (time)

Aseptic technique maintained

Total volume of ascitic fluid drained on insertion: (x) mL

5. Fluid samples sent (*please delete as appropriate*)

Cell count & differential // Culture & sensitivity // Protein // Albumin // Cytology (non-gynaecological specimen) // Amylase (if indicated) // Acid-fast bacilli

6. Post-procedure care

Monitor the drain output hourly

Drain to remain in for 6 hours or until 10 L drained max (whichever comes first)

Scheduled time for removal: (time)

Please document the total volume drained

7. For nursing staff

Observations every 30 mins for the first hour

Hourly thereafter

100 mL 20% HAS to be administered for every 2 L of ascitic fluid drained

Leave on free drainage

Monitor for bleeding

Clamp drained if SBP drops to <100 and inform the medical on-call team

Post-drain weight please

Please escalate to the medical staff if there are any concerns regarding the patient

Appendix C

What grade are you currently?

- Medical student

- FY1

- SHO

- Registrar

- SAS

- Consultant

Have you performed or assisted in an ascitic drain?

- Yes

- No

Do you know the list of equipment needed for an ascitic drain?

- Yes - All

- No - I know most (but not all) of the equipment needed

- No - I don’t know any of the equipment needed

Are you able to locate all the equipment needed?

- Yes - I know where to find each piece of equipment

- No -  I know where to find most (but not all) of the equipment needed

- No - I don’t know where to find any of the equipment

In how many different locations is the equipment kept in?

- 1

- 2-5

- 6-9

- >10

Who do you ask if you need help to locate the equipment?

- Junior doctor

- Registrar

- Consultant

- Healthcare assistant

- Band 5 Nurse

- Nurse in Charge

- Ward manager

If asking for help, on average, how many other healthcare professionals do you need to ask for help to locate the equipment?

- 1

- 2-3

- 4-5

- 5-6

- >6

How long on average does it take for you to locate and gather the equipment

- <5 minutes

- 6-10 minutes

- 11-15 minutes

- >15 minutes

Do you think a resource summarizing all the equipment needed would be useful for you?

- Yes

- No

- Unsure

Appendix D

What grade are you currently?

- Medical student

- FY1

- SHO

- Registrar

- Consultant

- Other

Have you seen the displayed poster on the equipment needed for the ascitic drain?

- Yes

- No

Do you think the poster is helpful in shortening the time needed to assemble the equipment to carry out an ascitic drain?

- Yes

- No

- I don’t know

Overall, do you find the posters useful?

- Yes

- No

Any additional comments?

Appendix E

Have you documented after an ascitic drain insertion?

- Yes

- No

How long does it take for you to document?

- 1-3 minutes

- 4-6 minutes

- 7-10 minutes

- >10 minutes

Do you need to ask for guidance from others when documenting?

- Yes

- No

Would a smart text with all the details be of use to you?

- Yes

- No

Appendix F

Do you find the ascitic drain smart text useful?

- Yes

- No

Do you think the smart text will shorten the time spent documenting?

- Yes

- No

How much time do you anticipate spending on documenting ascitic drain documentation with our next smart text?

- <1 minute

- 1-3 minutes

- 4-6 minutes

- 7-10 minutes

- >10 minutes

Do you anticipate still needing to ask others what to include in your ascitic drain insertion note? - i.e., on technique, instructions for nurses, when to remove

- Yes

- No

Please give any comments on how we can improve
